# Grade repetition among learners with and without disabilities in two provinces of South Africa

**DOI:** 10.4102/ajod.v14i0.1676

**Published:** 2025-08-21

**Authors:** Nicola Deghaye, Grace Leach

**Affiliations:** 1 Department of Research on Socioeconomic Policy, Faculty of Economic and Management Sciences, Stellenbosch University, Stellenbosch, South Africa; 2 Health Policy Unit, Department of Public Health, Institute of Tropical Medicine Antwerp, Antwerpen, Belgium

**Keywords:** learners with disabilities, grade progression, grade retention, inclusive education, education management information systems, low- and middle-income countries, disability

## Abstract

**Background:**

It is critical that disability-disaggregated indicators of educational outcomes are developed and monitored in low-and middle-income countries (LMICs) to demonstrate whether progress is being made towards educational equality.

**Objectives:**

To design, test and analyse new indicators of grade progression for learners with disabilities relative to learners without disabilities in South Africa. To determine which indicators are the most appropriate for future monitoring.

**Method:**

We undertook the first-ever quantitative analysis of grade repetition and age-for-grade of learners with disabilities relative to learners without disabilities using student-level data collected in the new Education Management Information System (EMIS). Using a longitudinal student-level dataset extracted from EMIS, we conducted cohort analyses of grade progression from 2017 onwards, disaggregated by gender and disability category.

**Results:**

On average, learners with disabilities experienced grade repetition more frequently than learners without disabilities and were older than their peers. Grade repetition rates decreased from 2017 to 2023 in mainstream schools in KwaZulu-Natal (KZN) province but increased in special schools. Comparatively, 54% of learners without disabilities who started Grade 1 in 2017 progressed to Grade 7 without repetition, versus 20% of learners with disabilities (Gauteng) and only 12% of learners with disabilities (KZN).

**Conclusion:**

The high rates of grade repetition among learners with disabilities suggest that reasonable accommodations and curriculum differentiation have not been fully implemented in schools.

**Contribution:**

There has been a substantial decline in reporting of learner disability status in Gauteng province since 2022 which warrants further investigation.

## Introduction

In recent years, there has been an increased focus on achieving educational equality between learners with and without disabilities and an increasing awareness of the need for disability-disaggregated data to monitor the exclusion of learners with disabilities in education systems. In South Africa, routine monitoring by the Department of Basic Education (DBE) has focused on enrolment of learners with disabilities and monitoring of selected inputs (DBE 2014a, 2014b, 2018a, 2020, 2021). Unfortunately, increased school enrolment in African countries is not translating into the expected learning outcomes (particularly reading proficiency) among primary school learners (Bold et al. 2017). For learners with disabilities, there is an even greater risk that school enrolment will not translate into learning unless reasonable accommodations are provided and teachers are adequately trained in inclusive education (Deghaye 2023). Consequently, it is critical that disability-disaggregated indicators of educational outcomes are developed and regularly monitored.

There are several ways to measure educational outcomes: learning outcomes (such as the proportion of learners who can read for meaning by age 10); educational attainment (highest level of education completed); and measures of school or grade progression, including rates of completion of primary or secondary schooling; rates of completion of school phases at an appropriate age; grade repetition rates (the percentage of learners who repeat a grade either over a period, a school phase or in a particular year); and proportions of children enrolled in school at an appropriate age.

Learning outcomes have been monitored in South Africa for more than a decade in large standardised international studies. However, these studies explicitly exclude learners with disabilities (LaRoche & Foy 2016; Schuelka 2013). Household surveys in South Africa measure disability well but do not measure foundational reading and mathematical proficiency. Thus, currently, it is impossible to evaluate learning outcomes, disaggregated by disability status, in South Africa. There is evidence that educational attainment is worse among adults with disabilities (Moodley 2017). However, measures of educational attainment are lagged variables and provide little information about the current generation of children with disabilities and the educational inequalities they face.

Researchers have called for monitoring of school progress of learners with disabilities (relative to learners without disabilities) in low-and middle-income countries (LMICs). Three sets of indicators have been proposed: (1) disability-disaggregated school drop-out; (2) disability-disaggregated grade repetition (Kuper, Saran & White 2020; Office of the United Nations High Commissioner for Human Rights 2020; Sprunt, Marella & Sharma 2016) and (3) the percentage of learners who are over-age for grade in primary and lower secondary schools, disaggregated by disability status (a Sustainable Development Goal [SDG] indicator). Household survey data indicate that rates of school drop-out among children with disabilities in South Africa were almost 50% by age 13 in 2011 (Mizunoya, Mitra & Yamasaki 2018:397).

Analysis of household survey data has shown that children with disabilities were over-represented among those who were currently repeating a grade in South Africa between 2011 and 2017 (McKenzie 2022). Household surveys, however, tend to underestimate grade repetition rates compared with school administrative data systems (Education Management Information Systems or EMIS). For example, Grade 1 repetition rates in the General Household Survey from 2014 to 2018 underestimated rates calculated in EMIS in 2016 by more than 50% in the general school population (Van der Berg et al. 2019b).

Indicators which estimate disability-disaggregated age-for-grade, capture the effect of two phenomena: over-age initial enrolment and grade repetition among learners with disabilities. Household survey data show that children with disabilities are more likely to be over-age for their current grade than children without disabilities. The only exceptions are children with *some difficulty seeing* or *some difficulty with walking and climbing stairs* (McKenzie 2022).

In this article, we provide the first ever analysis of grade repetition and age-for-grade of learners with disabilities relative to learners without disabilities in South Africa, using longitudinal student-level data collected in EMIS. This was made possible by the introduction of a student-level EMIS in South Africa, which covers special and mainstream schools and collects data on learner disability status. High rates of grade repetition among learners with disabilities may indicate that the curriculum is not accessible or that reasonable accommodation of learners’ individual needs is not being adequately provided.

We estimated various indicators of grade repetition, disaggregated by gender and disability category, as recommended by Kuper et al. (2020). We discuss which of these indicators is most appropriate and accurate, and make recommendations as to which indicators should be regularly monitored. This is the first time that cohort analysis has been applied to grade progression among learners with disabilities in South Africa.

## Background

### Inclusive education in South Africa

In South Africa, learners with disabilities may enrol in either ordinary (mainstream) or special schools. The key policy documents regulating inclusive education in South Africa are: White Paper 6 (National Department of Education 2001) and the Screening, Identification, Assessment and Support policy (DBE, 2014a). According to these policy documents, all learners, including learners with disabilities, should have access to four support programmes: (1) curriculum differentiation; (2) specialist staff; (3) specialised or adapted learning, and teaching support material and assistive technology; and (4) training of school personnel. Curriculum differentiation refers to the process of modifying, adapting, extending and varying teaching methods and strategies, assessment strategies and the content of the curriculum so that learners with different levels of functioning can learn effectively (DBE 2014; 2014a). The intensity and frequency of support would be higher in special schools, but learners enrolled in mainstream schools should also have access to low-frequency, low-intensity support from all four programmes. There is growing evidence of large gaps in provision of the four programmes of support (Deghaye 2023; Equal Education Law Centre 2022; Human Rights Watch 2015; Kelly & McKenzie 2018; McKenzie et al. 2020, 2021; Watermeyer et al. 2016).

School- and district-based support teams provide critical support to class teachers in identifying learners with disabilities and in designing and delivering interventions for these learners (DBE 2014a). The services provided by the school- and district-based support teams and special school resource centres should enable learning environments in mainstream schools to become accessible and enable schools to provide reasonable accommodation for learners, where required (Deghaye 2023). Reasonable accommodation refers to: necessary and appropriate adjustments and modifications that do not impose a disproportionate or undue burden, where needed, in a particular case, to ensure that children with disabilities are able to enjoy their rights (to education) on an equal basis with others (Deghaye 2023; United Nations 2007).

However, in 2017, school-based support teams existed in 99% of Gauteng schools and 62% of KwaZulu-Natal (KZN) schools. Comparatively, 81% of school-based support teams in Gauteng province received support from the district versus 61% in KZN province (Deghaye 2021). These gaps in support are likely to make identification of invisible learner disabilities difficult in many schools in KZN province.

The Screening, Identification, Assessment and Support (SIAS) policy (2014) further lays out a process for identifying learners in need of extra support or reasonable accommodation. The first step in the process of either accessing reasonable accommodation (or obtaining a disability label in the school system) is the completion of a *learner support-needs assessment* by the school-based support team. While 80% of schools in Gauteng were able to complete at least one *support-needs assessment* in 2017, only 43% of KZN schools were able to do so (author’s own calculations, School Monitoring Survey 2017). This suggests that, at the start of the analysis period (2017), more than half of KZN schools were not in a good position to undertake the process by which learners obtain access to reasonable accommodations or obtain a disability label in the school system. Disability status may only be captured in EMIS once several additional steps are completed and a health professional has confirmed that the learner has a disability (as defined by a set of criteria that are outlined in the SIAS policy, which include level of functioning and medical criteria).

As a result of the various implementation gaps outlined in this section, teachers are not being supported to provide reasonable accommodation of diverse learning needs. For example, the literature shows that the learning needs of learners with sensory disabilities and severe intellectual disability are not being accommodated adequately in schools (Human Rights Watch 2015).

There have been substantial developments in education policy for learners with intellectual disabilities (ID) since 2017. The identification of ID has improved substantially in special schools over the analysis period (personal communication, T. Levin, DBE). Two new (draft) curricula were released for public comment: a learning programme for children with severe to profound ID (in 2016) and a differentiated curriculum (Grade R to 5) for learners with mild to severe ID who can manage primary school-level content. These curricula must be finalised before they can be fully rolled out. Once implemented, there should be no grade repetition for learners who are following these curricula as each student should be following an individual support plan. Severe ID (SID) years were introduced from 2018 in Gauteng province, and from 2020 in KZN province. Severe ID years have also been introduced in the Eastern Cape, Mpumalanga and the North West province.

Overall, South Africa has a fairly well-developed policy promoting education of learners with disabilities (Grimes et al. 2023). Unfortunately, except for learners with SID, these policies have not yet been funded (Deghaye 2023; Equal Education Law Centre 2022; Financial and Fiscal Commission 2020).

### Grade repetition in South African schools

Grade repetition is meant to allow learners who have not met grade expectations a second opportunity to master the content of that grade and catch up with their peers (Kika & Kotze 2019). Despite being widely used, the evidence of its effectiveness is mixed (Kika & Kotze 2019; Van der Berg et al. 2019b). Recent evidence suggests that, in the general learner population in South Africa, repetition of Grade 1 is associated with some improvement in reading scores, but that Grade 2 and Grade 3 repetition are less effective. Learners who repeat an early grade continue to have much flatter learning profiles than progressed learners (Wills 2023).

Grade repetition rates in South Africa are high relative to other LMICs. In the general learner population, repetition rates are higher in Grade 1 and Grade 4 than in other primary school grades, and are higher in secondary than in primary school, reaching a peak in Grade 10 (Van der Berg et al. 2019b). Education policy stipulates that a learner may only repeat a grade once and only once within each schooling phase (Grades 1 to 3; Grades 4 to 6; Grades 7 to 9 and Grades 10 to 12) (DBE 2012; Department of Education 1998). While grade repetition rates among learners without disabilities have reduced over time, the progression policy has not been implemented universally. The progression policy emphasises that districts and schools must have clearly articulated intervention strategies including early identification of learners at-risk of grade repetition. The school, district and province must develop and implement additional learning opportunities targeted at these learners (Kika & Kotze 2019).

In 2020 and 2021, grade repetition rates declined substantially, because of coronavirus disease 2019 (COVID-19) policy decisions to reduce assessment, cancel examinations and trim the curriculum (Van der Berg et al. 2023a; Wills & Qvist 2023). In this analysis, we expected to find improved grade progression in 2020 and 2021 among learners with and without disabilities.

## Research methods and design

Descriptive statistical analysis was used to estimate various indicators of grade repetition, and age-for-grade for learners with and without disabilities using school administrative data collected from all schools in Gauteng and KZN provinces who reported in the South African School Administration Monitoring System (SA-SAMS) from 2017 to 2023.

### Ethical considerations

We received approval to conduct secondary analysis of longitudinal student data from Stellenbosch University’s Social, Behavioural and Education Ethics Committee (ECO-2022-25130) and via an amendment (SBE-2024-25130). The project was classified as a low risk project as it involved analysis of secondary data and there was no interaction with any learners during the course of the research. Details of any informed consent that was provided by learners were not available to the researchers.

The dataset is managed by the Michael and Susan Dell Foundation (MSDF). The raw data are collected quarterly from schools via SA-SAMS and is housed in the Data Driven Districts (DDD) operational data store. The provincial departments of education provided written permission for MSDF to release the anonymised data, for the purposes of this research project.

### Data description

In 2017, 81% of schools in Gauteng (2535 schools) and 81% of schools in KZN (4910 schools) reported at least some data in DDD dataset. We are confident that the sample of schools in the dataset is representative of (public-sector) mainstream and special schools in the two provinces. The sample includes some independent schools but under-represents them.

Each learner is identified by an anonymised unique student identifier, which was used by MSDF to link learner data from 2017 to 2023 to create a longitudinal student-level dataset, which we refer to as the DDD longitudinal dataset. The dataset includes student-level data on learner age, grade, gender and disability status. The dataset has previously been used to describe learner progression and grade repetition of learners in ordinary grades (regardless of disability status) (Van der Berg et al. 2019a).

All implausible or incomplete duplicate student records were dropped from the dataset. Remaining duplicate records were dropped at random. We combined SID years and ordinary grades to allow inclusion of all learners with ID. For example, learners in SID year 1 and in ordinary grade 1 are grouped as *Grade 1* learners. Because the dataset includes the grade of each student in each year, we could tag whether a student was repeating a grade in any year. We tagged the student identifier each time a learner repeated a grade. All analysis was conducted in RStudio 4.4.1 (R Core Team 2024).

#### Data on disability status

A total of 40 categories of disability were recorded in the DDD longitudinal data. We grouped these categories into four broad disability categories to allow large enough sub-groups for meaningful analysis: learners with: (1) mild to moderate or (2) severe to profound intellectual disability; (3) specific learning disabilities (SLD) and (4) *other disabilities*, as shown in Table 1-A1. The *other disabilities* category is a very broad category which includes all learners whose primary disability is not an intellectual or learning disability. Learners in this group may, in fact, have intellectual or learning disabilities in addition to their primary disability.

The disability categories in the EMIS data do not match the domains of disability in the assessment forms used by district-based support teams and health professionals since 2014. The categories in EMIS are often based on the presence of a medical condition, while the process of identification in the school system is largely centred on functioning and is aligned with the biopsychosocial model of disability. This introduces a possible source of data error as there is no simple way of matching the categories of disability in EMIS with those on the assessment forms. In many cases, the data clerk must make a fairly random choice of disability type.

*Mild to moderate ID* was the most frequently identified disability type in Gauteng (43% of learners with disabilities or 18 429 learners in 2019), while *other disabilities* were most commonly identified in KZN (10 910 learners or 37% of learners with disabilities). In 2019, 69% and 66% of learners with disabilities in Gauteng and KZN (respectively) were male. This aligns with trends previously observed in EMIS data and household surveys in South Africa (Deghaye 2023) and international evidence of higher rates of intellectual disability among male learners (Maulik et al. 2011).

### Completeness of data on disability status

In KZN, 64% of schools (3895 schools) reported the enrolment of learners with disabilities each year from 2017 to 2023. In Gauteng, from 2017 to 2021, between 58% and 61% of schools (approximately 1900 schools) reported enrolment of learners with disabilities. This declined to 53% in 2022 and 34% (or 1053 schools) in 2024. In the same period, the reported number of learners with disabilities in Gauteng province declined sharply from approximately 44 000 learners in 2017 to approximately 28 000 in 2023.

This decline was driven by a steady decline in reporting in mainstream schools (shown in Table 1, column 1). Special school enrolment, by contrast, was fairly stable over the period. In KZN, the reported number of learners with disabilities in mainstream primary schools in 2023 was low, as shown in Table 1, but reporting was fairly stable for the rest of the period.

**TABLE 1:** Percentage of learners with disabilities currently repeating a primary school grade, by school type.

Years	Mainstream schools				Special schools			
	Gauteng		KwaZulu-Natal		Gauteng		KwaZulu-Natal	
	*n*	Repeating (%)	*n*	Repeating (%)	*n*	Repeating (%)	*n*	Repeating (%)
2018	18 951	23	20 003	23	7854	35	2775	29
2019	17 100	25	20 824	17	7642	37	2905	31
2020	14 432	27	20 424	18	6582	40	2985	36
2021	11 168	26	19 091	12	6737	42	3054	55
2022	7755	30	17 036	17	6496	51	2739	46
2023	5275	30	13 629	16	6766	51	2730	47

Note: DDD longitudinal dataset, all learners in Grade 1 to 7 in public sector schools.

**FIGURE 1:**
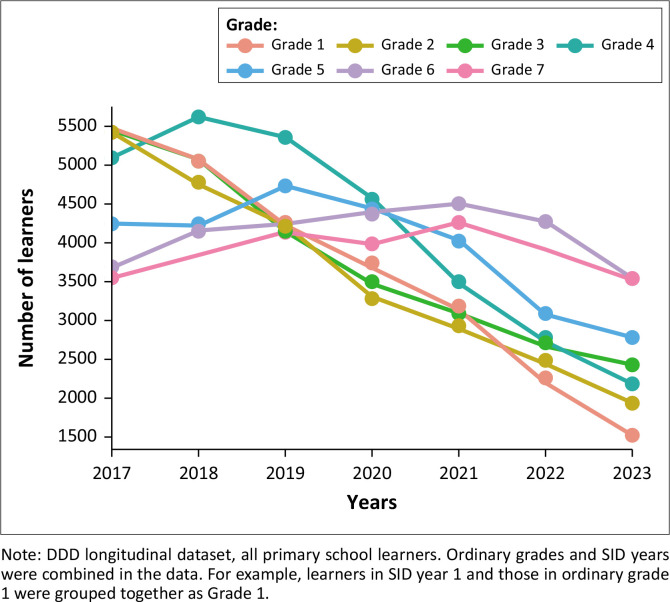
Reported number of learners with disabilities, by grade: Gauteng province.

**FIGURE 2:**
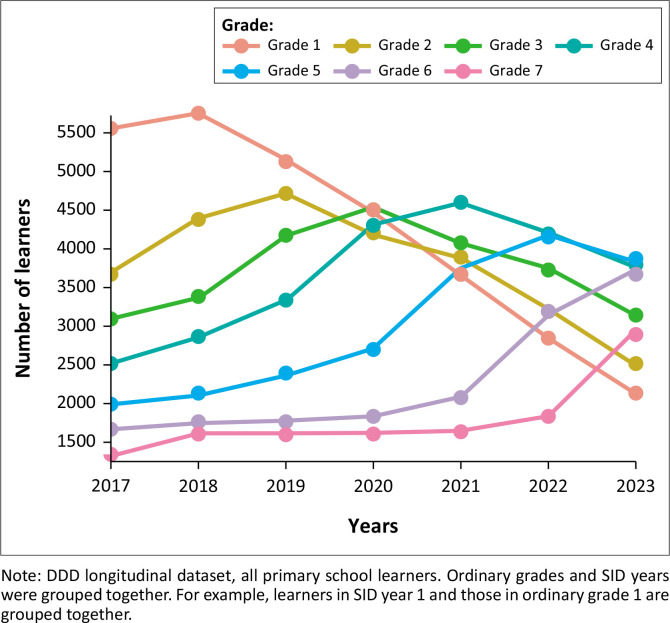
Reported number of learners with disabilities, by grade: KwaZulu-Natal province.

In Gauteng province, the decline in reporting was particularly strong among learners with mild to moderate ID (whose numbers showed a steady decline over the period, such that they almost halved from 2017 to 2023) and among Grades 1 to 3 learners (shown in Figure 1 and Figure 2).

The drastic decline in enrolment of learners with disabilities observed in Gauteng province, particularly in the early grades, is not credible. The reporting problems identified in this study are reported in more detail elsewhere (Deghaye et al. forthcoming) and deserve more investigation. From 2021 onwards, the data are unlikely to represent all learners with disabilities enrolled in schools in Gauteng, but rather all those whose disability status has been identified and reported and should be interpreted as such. Because of the reporting problems identified in Gauteng province, we have largely focused on the results in KZN province.

### Methodology

Our goal was to produce several new indicators of grade repetition, and age-for-grade for learners with and without disabilities which could be produced by the DBE in the future for internal monitoring and external reporting. Descriptive statistics are better suited for this purpose than multivariate analysis.

#### Descriptive analysis

We conducted student-level, cross-sectional analyses of overall grade repetition patterns for the entire population of learners (all learners, in all grades) over the full period of analysis (2017 to 2023) in KZN and Gauteng provinces.

The accuracy of the descriptive analysis may be biased by increasingly incomplete reporting of disability status in Gauteng province from 2022 onwards. No statistical tests were conducted to determine whether the observed differences by disability status were statistically significant as the possible selection bias in the data may make these results misleading.

#### Cohort analysis

We used cohort analysis techniques previously developed by Van der Berg et al. (2019a, 2021, 2023b) and applied these techniques (with some modification) to disability-disaggregated data to track learner flows through primary and secondary school.

Typically, disability status appears to be entered when the learners first enrolled in a school. We analysed one cohort who started Grade 1 in 2017, and three cohorts who started Grade 8 in 2017, 2018 and 2019, respectively. Between 2017 and 2019, reporting of disability status appears to be more widespread in Gauteng province than in 2022 and 2023. As the cohort analysis is limited to learners who were identified as disabled before 2019, it is unaffected by incomplete reporting observed in 2022 and 2023 (and thus more representative of the whole population of learners with disabilities).

As the student identifier in SA-SAMS is unique within but not across schools, learners can only be tracked in the cohort analysis if they do not change schools in the analysis period (Van der Berg et al. 2019a). Thus, the cohort analysis follows all learners who remain in the provincial school system and do not change schools during the analysis period (two thirds of students who started in Grade 1 in 2017). This has two implications: (1) the cohort is not fully representative of the full student population in Gauteng or KZN and (2) we cannot track learners from Grade 1 to Grade 9 as most learners change schools when they start secondary school (Grade 8). This is unfortunate as school progression to the end of Grade 9 is a key indicator for SDG 4.5. Learners with disabilities change schools at roughly the same rate as learners without disabilities (roughly one in three students changed school in the 7-year period in which we observed them). Thus, we believe that cohort analysis of school progression, disaggregated by disability status, is robust.

To perform the Grade 1 cohort analysis, we restricted the dataset to those children who started Grade 1 in 2017. This year was selected as the starting point for the cohort analysis as by 2017, the new EMIS was in widespread use and most teething problems associated with the system change had been overcome. The period 2017 to 2023 represents the first 7-year period for which data was available to follow a cohort of learners from Grade 1 to the point where they should have started the final year of primary school (Grade 7) if they did not repeat a grade. It is unfortunate that this period includes 2 years where schooling was dramatically affected by COVID-19.

## Results

### Descriptive statistics (full population of learners)

The primary school grade distribution of learners with disabilities changed substantially from 2017 to 2023 in both the provinces (shown in Figure 1 and Figure 2). There is a consistent and substantial decline in the number of Grade 1 to 3 learners with reported disabilities in Gauteng province from 2017 to 2023 (shown in Figure 1). In KZN, the same decline is seen in Grade 1, but not in Grades 2 or 3 (shown in Figure 2). In KZN, there were strong increases in the number of learners with disabilities in Grades 5, 6 and 7 after 2020. This suggests reduced dropout or improved grade promotion after 2020 in KZN, in line with the general learner population (Van der Berg et al. 2023).

In Gauteng province, *some* of the reduced enrolment in the early grades could be explained by the introduction of SID years from 2017 onwards. By 2023, up to 2000 learners were enrolled in SID years 4 or higher. Many of these learners may previously have repeated Grades 1 to 3 multiple times. The much lower number of learners with disabilities in Grade 1 over time (shown in Figure 1 and Figure 2) is more likely the result of reduced identification of disability among newly-enrolled Grade 1 learners in later years. Alternatively, there could have been a slump in reporting of disability status in EMIS upon initial enrolment in school (Grade 1) from 2020 as school administrators became overwhelmed by additional responsibilities brought about by the COVID-19 epidemic.

Table 1 shows that the percentage of learners with disabilities who were currently repeating a primary school grade declined in mainstream schools in KZN from 2017 to 2023. Improvements in progression in mainstream schools from 2020 were probably because of more lenient grade progression in 2020 and 2021 (Van der Berg et al. 2023a; Wills & Qvist 2023). However, rates of repetition increased substantially over time in special schools. Approximately half of all learners in special schools in KZN (and Gauteng) were currently repeating a grade each year from 2020 to 2023. This is alarming and suggests that the COVID-19 disruption dramatically disrupted grade progression in special primary schools. Grade repetition at this scale will lead to longer waiting times for admission into special schools and larger class sizes (which were already unacceptably large) (Equal Education Law Centre 2022).

Only learners with high-level support needs should be enrolled in special schools, while learners with disabilities who are enrolled in mainstream schools could have low-, moderate- or high-level support needs. Thus, the higher repetition rates in special schools are likely driven by the higher average level of support among learners with disabilities enrolled in that setting. Even so, the current repetition rate in special schools appears to be excessively high.

**FIGURE 3:**
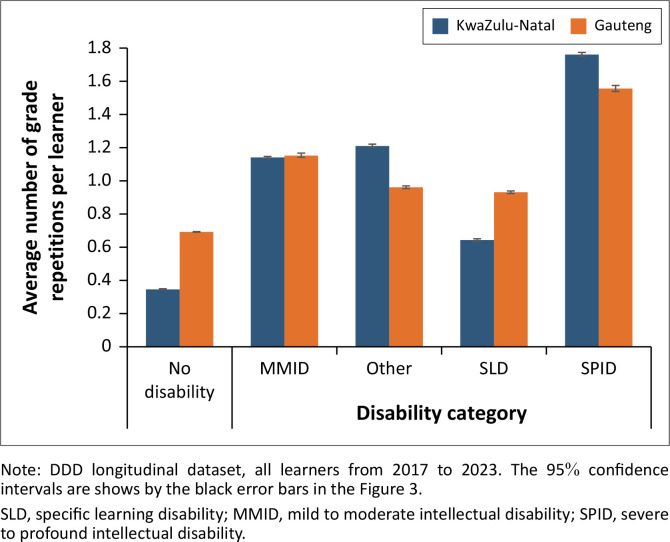
Average number of grade repetitions per learner by disability category.

Figure 3 demonstrates that, from 2017 to 2023, the average learner with disabilities experienced grade repetition more frequently than the average learner without disabilities. Grade repetition rates differ substantially by disability category and the patterns are fairly consistent between provinces. On average, learners identified with SLD repeated less frequently than learners with *other disabilities.* Learners with severe to profound ID experienced the highest average number of grade repetitions.

The average number of grade repetitions over a period or the percentage of learners currently repeating a grade does not show how grade repetition changes as a child moves through the different levels of schooling. To address this, we examined the frequency of grade repetition for each primary school grade in the period 2017 to 2023.

Table 2 shows the number of learners who repeated each primary school grade once, twice and up to five times, and what proportion of repeaters are identified as having a disability. In both provinces, there is a tendency for learners with disabilities to be over-represented among learners who repeat grades multiple times. In KZN, learners with disabilities make up 1.2% of all learners, yet represent 5.0% to 6.0% of learners who repeat early grades twice and up to 19.0% of learners who repeat a grade 3 times. It is disturbing to find evidence of learners repeating a single grade four or five times. In Gauteng, learners with disabilities make up a very high proportion of learners who repeat an early grade more than twice.

**TABLE 2:** Frequency of grade repetition, disaggregated by grade of enrolment: KwaZulu-Natal.

Grade repeated	Repeaters	No. of times a learner repeats the grade				
		1	2	3	4	5
Grade 1	Number	164 796	16 351	2073	278	34
	% with disabilities	3	6	9	16	29
Grade 2	Number	107 407	9256	1136	125	22
	% with disabilities	3	5	7	6	18
Grade 3	Number	89 904	7321	814	94	9
	% with disabilities	3	6	9	16	67
Grade 4	Number	111 068	8786	1075	119	21
	% with disabilities	4	5	9	8	19
Grade 5	Number	75 628	5878	720	98	5
	% with disabilities	3	7	19	28	20
Grade 6	Number	57 027	3678	407	39	2
	% with disabilities	2	3	3	0	0
Grade 7	Number	57 320	3878	389	39	9
	% with disabilities	2	4	3	3	0

*Note:* DDD longitudinal dataset (KwaZulu-Natal), all learners in all primary school grades from 2017 to 2023.

Sustainable Development Goal Indicator 4.5.1 calls for monitoring of the percentage of children who are over-age for grade in primary and lower secondary schools, disaggregated by disability status. This indicator captures the effect of late enrolment in school and grade repetition. To test whether this could be calculated in the DDD longitudinal dataset, we examined the age profile of learners with and without disabilities, by grade.

**FIGURE 4:**
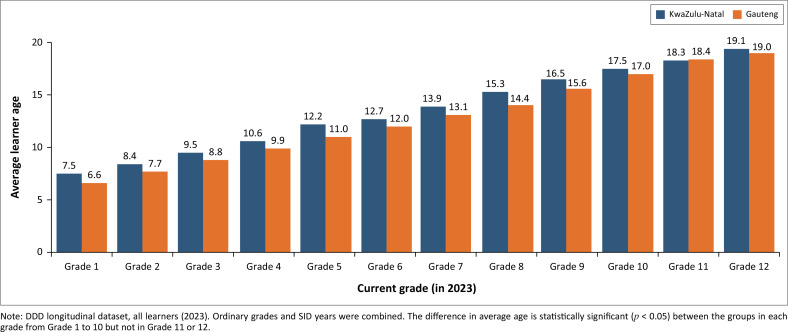
Average learner age by grade: KwaZulu-Natal, 2023.

Figure 4 shows that in KZN in 2023, learners with reported disabilities were, on average, significantly older than learners without disabilities in the same grade. In 2023, the difference is greatest in Grade 5 (12.2 months). The disability-related differences in age persist until Grade 10. This suggests that over-age learners with disabilities tend to leave the school system in Grade 9 or 10. Thus, from Grade 10 onwards this is not an appropriate disability-disaggregated indicator of educational outcomes. The SDG Indicator’s focus on primary and lower secondary school is appropriate in South Africa. The indicator was calculated for Gauteng province, and the average age for grade was found to be increasing over time. For example, the average age in Grade 1 (learners with disabilities) increased from 8.7 years in 2018 to 10.1 years in 2023. We suspect the average age of learners with disabilities is being skewed upwards by incomplete reporting of disability status in 2022 and 2023. As we are uncertain about the accuracy of these results, they are not shown.

### Cohort analysis

**FIGURE 5:**
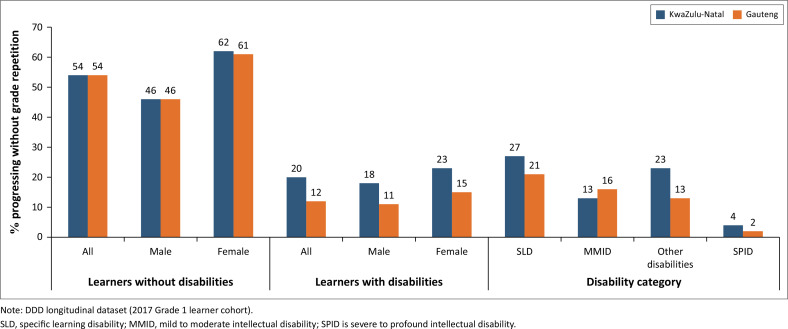
Percentage of learners progressing from Grade 1 to Grade 7 without grade repetition: 2017 to 2023.

Given that the reporting in Gauteng province in 2017 was much more complete than in later years, we have reported the results of the cohort analysis in both provinces. Figure 5 shows that 12% and 20% of learners with disabilities who started Grade 1 in Gauteng and KZN in 2017, respectively, progressed to Grade 7 without repetition, compared with 54% of learners without disabilities in both the provinces. This is a substantial difference. In both provinces, fewer male learners progressed to Grade 7 without repetition than females, regardless of disability status. Figure 5 demonstrates that there are substantial differences in grade progression by disability category. A higher proportion of learners with SLD progressed to Grade 7 than learners with other disability types. An extremely low percentage of learners with severe to profound ID progressed through primary school without repeating a grade.

Finally, we analysed grade progression in three secondary school cohorts. Table 3 shows that, in Gauteng province, only 10% of the learners with disabilities in the 2017 Grade 8 cohort progressed to Grade (or SID year) 12 by 2023, compared to 36% of learners without disabilities. In KZN, in the 2017 Grade 8 cohort, the differences in progression rates between learners with and without disabilities are less pronounced, but still substantial (23% versus 32%). In both provinces, progression rates to Grade 12 vary substantially between the 2017 and 2019 cohorts. Grade progression improves in Gauteng in later cohorts, for learners with and without disabilities. In KZN, however, grade progression worsens for learners with disabilities in the later cohorts, suggesting that they did not benefit from more lenient grade progression policies in response to the COVID-19 crisis.

**TABLE 3:** Percentage of learners progressing from Grade 8 to 12 without (further) repetition.

Learner disability status	2017–2021 cohort		2018–2022 cohort		2019–2023 cohort	
	*n*	% progressing	*n*	% progressing	*n*	% progressing
**Gauteng**						
Without disabilities						
All	140 052	36.0	150 538	42.0	163 132	42.0
Males	71 760	28.0	76 637	33.0	83 821	33.0
Females	68 292	44.0	73 901	52.0	79 311	52.0
With disabilities						
All	3328	10.0	3385	20.0	2679	14.0
Males	2327	8.0	2300	15.0	1896	12.0
Females	1001	13.0	1085	31.0	783	20.0
By disability category						
SLD	557	17.0	704	50.0	515	29.0
MMID	1883	4.0	1826	7.0	1555	7.0
SPID	226	3.0	155	1.0	101	1.0
Other	662	22.0	700	30.0	508	26.0
**KwaZulu-Natal**						
Without disabilities						
All	163 353	32.0	175 733	37.0	184 541	36.0
Males	87 203	25.0	92 618	28.0	97 728	28.0
Females	76 150	41.0	83 115	46.0	86 813	46.0
With disabilities						
All	1052	23.0	963	16.0	1167	20.0
Males	628	14.0	634	11.0	748	15.0
Females	424	35.0	329	26.0	419	29.0
By disability category						
SLD	292	15.0	284	9.0	297	16.0
MMID	240	8.0	250	2.0	284	2.0
SPID	27	-	33	-	46	-
Other	493	35.0	396	31.0	540	33.0

*Note:* DDD longitudinal dataset (Grade 8 learner cohorts: 2017, 2018, 2019). The *n* represents the number of learners who started Grade 8 in the first year (2017, 2018, 2019). Learners may have repeated a grade in primary school. Learners in the 2017 cohort may be repeating Grade 8 in 2017.

SLD, specific learning disability; MMID, mild to moderate intellectual disability; SPID, severe to profound intellectual disability.

In KZN province, the subgroup of learners with severe to profound ID is too small for meaningful analysis, and results are not shown. More fundamentally, many learners with ID are likely to progress to technical or vocational education after Grade 9, thus measuring progression to Grade 12 is inappropriate.

## Discussion

This research provides the first-ever evidence of grade repetition among learners with disabilities in South Africa and provides new school-level estimates of age-for-grade among learners with disabilities, relative to those without disabilities.

We have shown that rates of grade repetition are higher among learners with reported disabilities than among learners without disabilities in KZN and Gauteng provinces in the period 2017–2023. We have also shown that in KZN, in each grade up to Grade 9, learners with reported disabilities are, on average, older than learners without disabilities. These results align with previous research which used household survey data from 2011 to 2017 (McKenzie 2022). Incomplete reporting in Gauteng in recent years meant that some of the full-sample indicators produced unreliable results.

Despite the flaws in the data on disability category in EMIS, the analysis shows that grade repetition is higher among learners with intellectual disabilities than among learners who are reported to have SLD or *other disabilities*. Again, this confirms McKenzie’s (2022) finding that there are differences in school progression by domain of disability.

Learners with reported disabilities (of any type) are over-represented among learners who repeat a grade more than once. This finding suggests a priority point for disability screening. Every student who is repeating a grade and is still not meeting grade-level expectations must be screened using the processes outlined in DBE’s SIAS policy (2014). Given that few childhood disability screeners have been validated for use by teachers, we suggest that the teacher version of the Washington Group Child Functioning Module could be used as a screening tool to complement the existing screening process. The Child Functioning Module guides teachers to consider and identify functional difficulties a child may be experiencing in seeing, hearing, mobility, fine motor skills, communicating, learning, remembering, concentrating, controlling their behaviour, coping with change, forming relationships and with anxiety and depression (Cappa et al. 2018). This tool should be piloted among South African teachers.

In some instances, grade repetition may be an appropriate remediation strategy. But to be successful, it must be linked with the provision of reasonable accommodation for learners with disabilities. The grade repetition rate among learners with disabilities is currently unacceptably high. These findings suggest that there has been over-reliance on grade repetition as a form of remediation among learners with disabilities. These results align with existing literature which suggest that many learners with disabilities are not receiving reasonable accommodation (Human Rights Watch 2015) and that teachers are not receiving enough training or support to provide adequate accommodation to learners with disabilities (Deghaye 2021; Kelly & McKenzie 2018). Over-reliance on grade repetition is likely linked to the South African government’s failure to fully fund and implement the four programmes of support outlined in the SIAS policy. For example, additional posts for specialist staff in district offices, which are promised in district norms, are only being created incrementally from 2024 onwards for reasons of affordability (DBE 2018b). Creating the promised posts in district offices would enable additional support to be provided to school-based support teams and (indirectly) to learners at risk of grade repetition.

According to the progression policy, districts and schools should have clear intervention strategies and provide additional learning opportunities for learners at risk of repetition. Our findings suggest that learners with disabilities are not being fully considered in these strategies or are not receiving the reasonable accommodations they need. The progression policy may need to be better integrated with the SIAS policy to ensure that teachers consider reasonable accommodation and curriculum differentiation when developing intervention strategies to prevent grade repetition.

Grade repetition is costly as it increases the number of years that a student spends in school (Van der Berg et al. 2019b). The direct cost of an additional learner-year in 2024 is ZAR 1748 or USD 95 (the per-learner allocation paid to schools by government). The direct cost of an additional learner-year in a special school is much higher (ZAR 7021 or USD 426 per learner in 2020/1 as Equal Education Law Centre (2022). Thus, even if one considers only the direct cost of repetition, repetition rates of nearly 50% in special schools are costly to the state.

We suggest that additional teacher training on curriculum differentiation and provision of reasonable accommodations, especially during assessment, could reduce reliance on repetition. Finalising and implementing the draft curricula for learners with ID should also lead to improvements in grade progression. The cost of these interventions could be covered in part by cost savings realised through reduced grade repetition.

These results suggest that, before the introduction of the new curricula, most learners with severe to profound ID experienced multiple grade repetitions in primary school. This finding demonstrates the need for the new curricula proposed for this group of learners. The new curricula should result in reduced grade repetition among learners with ID as learners would follow individual education plans. The differentiated curriculum for learners with mild to severe ID must be finalised so that it can be widely implemented. Once it is implemented, the 2017 cohort of Grade 1 learners with ID will form a useful baseline against which to measure the impact of the new curricula on grade progression.

### Recommendations for future monitoring and research

The research further aimed to determine which of the estimated indicators were most appropriate and accurate, and to make recommendations as to which indicators should be regularly monitored.

As grade repetition is not uniform across grades, and grade-distribution of learners with reported disabilities was shown to be changing substantially over the analysis period, it is difficult to interpret indicators that summarise the experience of the average learner over time (such as the average number of grade repetitions per learner, shown in Figure 3, or the percentage of primary school learners who are currently repeating a grade, as shown in Table 1). Further, because the data were only available for 7 years instead of the full 10 years of compulsory schooling, these two indicators may underestimate grade repetition over the whole compulsory school-going period.

Grade-specific indicators (such as the average learner age per grade, disaggregated by disability status, shown in Figure 4, or the frequency of grade repetition per grade, shown in Table 2) are more useful as they are unaffected by changes in the grade-distribution over time. However, these indicators were shown to be highly sensitive to incomplete reporting in the Gauteng data.

As discussed in the methodology section, the cohort analysis we conducted provides more accurate indicators of grade progression than other indicators presented in this article. Therefore, we recommend cohort analysis as the best descriptive indicator of disability-disaggregated learner progress.

Given that just over half of all learners without disabilities are able to progress to Grade 7 without grade repetition, it is unrealistic to expect learners with disabilities (who are often at a tremendous disadvantage to their peers) to do so. Instead, we suggest that two modified indicators are regularly tracked:

- The proportion of learners with disabilities who progress from Grade 1 to 7 with one (or zero) grade repetitions, relative to learners without disabilities.
- The proportion of learners with disabilities who progress from Grade 1 to 7 with two (or fewer) grade repetitions, relative to learners without disabilities.

These indicators could be estimated for the 2017 cohort using 2024 and 2025 EMIS data, once it becomes available. The indicator should be disaggregated by gender and category of disability and should ideally be reported separately for special and mainstream schools. This is important, as the very high rates of grade repetition found in special schools require close monitoring. The cohort analysis should be conducted in all provinces.

We recommend that a foundation phase cohort analysis be undertaken to provide a timely assessment of how COVID-19 impacted grade progression of early-grade learners with disabilities. The 2017 Grade 1 cohort represents the last cohort to (potentially) complete foundation phase before the COVID-19 disruption. A foundation phase cohort analysis is also critical for investigating whether reduced enrolment of learners with disabilities in Grade 1 (shown in Figure 1) is because of reduced repetition in the early grades after 2017.

The Grade 8 cohort analysis may not be appropriate for learners with ID as it is often appropriate that learners with ID move from the academic school curriculum to technical or vocational education after Grade 9. Average learner age at the beginning of Grade 9 is the most appropriate indicator for monitoring grade progression for learners with ID. Sustainable Development Goal Indicator 4.5.1 is thus an appropriate indicator at Grade 9 level.

For all other learners, we recommend that the secondary school cohort analysis be slightly adjusted to estimate:

- The proportion of learners who progress from Grade 8 to 12 with one (or zero) grade repetitions, relative to learners without disabilities
- The proportion of learners who progress from Grade 8 to 12 with two (or fewer) grade repetitions, relative to learners without disabilities.

When interpreting the data on grade progression in secondary school, one must consider that learners with disabilities who progress to secondary school are a positively selected group. They may represent learners with disabilities who have the most supportive families, have attended the more inclusive schools or have the highest levels of functioning. These factors cannot be measured in EMIS and some of them cannot be influenced by the school system.

Unfortunately, incomplete reporting of student disability status in Gauteng in recent years will reduce the accuracy of cohort analysis in cohorts that started Grade 1 or 8 from 2021 onwards unless data quality is addressed.

Disability-disaggregated school drop-out has been recommended as a useful measure of educational outcomes (Kuper et al. 2020). However, there is a risk of overestimating school drop-out rates in EMIS data. Household survey data should be used to estimate school drop-out, as done previously by Mizunoya et al. (2018).

The grade-distribution of learners with disabilities is shown to be changing over the period, with intermediate phase enrolment trending upwards over time and foundation phase enrolment trending downwards in KZN. These trends align with Census 2022 data which show that the percentage of people with disabilities over the age of 20 who had completed primary school trended upward from 2011 to 2022 (Statistics South Africa 2024). Grade 1 enrolment of learners with disabilities has, however, been declining in KZN since 2018. The reasons for this are not yet clear and require more investigation.

Finally, we recommend that multivariate analysis of the KZN data be conducted to identify factors which interact with disability to make certain groups of learners particularly vulnerable to grade repetition.

### Limitations

The indicators estimated in this study are not internationally comparable as identification of disability in EMIS is different to that in all other school systems, except Namibia (MiET, Africa Disability Alliance and Differences 2014). The disability status indicator in EMIS is narrowly- and medically-defined, and is likely to under-identify student disability (Deghaye 2023; Dube & Mont 2021). We suspect, for example, that there are a large number of learners with unidentified disabilities among those learners who repeat a grade twice or more (shown in Table 2). Further investigation of the Gauteng data on learner disability status is needed.

## Conclusion

This study provides powerful new evidence that grade repetition continues to be widely used as a remediation strategy for learners with disabilities. At times, grade repetition may be appropriate and effective for learners with disabilities. However, the current rates of grade repetition among learners with reported disabilities are unacceptably high and suggest that reasonable accommodations and curriculum differentiation have not been fully implemented in schools.

We propose that the proportion of learners with disabilities who progress from Grade 1 to 7 with one or fewer grade repetitions and the proportion of learners with disabilities who progress from Grade 1 to 7 with two or fewer grade repetitions should be adopted as indicators of disability inclusion in schooling. These indicators should be monitored annually relative to learners without disabilities.

## Appendix 1

**TABLE 1-A1:** Grouping of education management information system disability categories used in analysis.

Broad category used in the analysis	Raw disability category in EMIS
Severe to profound intellectual disability	Profound intellectual disability
	Severe intellectually disabled
Mild to moderate intellectual disability	Mild to moderate intellectual disability
	Moderate to severe intellectual disability
Specific learning disorder	Dyscalculia
	Dysgraphia
	Dyslexia
	Attention deficit disorder
	Attention deficit hyperactive disorder
	Auditory processing disorder
	Autistic spectrum disorder
	Cerebral palsy
	Dysphasia
	Epilepsy
	Foetal alcohol syndrome
	Traumatic head injury
	Blind
	Partially sighted
	Conduct disorder
	Deaf
	Hard of hearing
	Deaf/blind
	Chronic medical condition
	Hemiplegic
	Impaired lower limb
	Impaired upper limb
	Paraplegic
	Quadriplegic
	Anxiety disorder
	Depression
	Bipolar
	Insomnia
	Psychiatric disorder
	Schizophrenia
	Mental illness: Other
	Obsessive compulsive disorder (OCD)
	Social communication disorder
	Speech sound disorder
	Stuttering
	Aphasia

EMIS, education management information system.
